# Transforming the systematic review service: a team-based model to support the educational needs of researchers

**DOI:** 10.5195/jmla.2018.430

**Published:** 2018-10-01

**Authors:** Stephanie Clare Roth

**Affiliations:** Biomedical and Research Services Librarian, Ginsburg Health Sciences Library, Temple University, Philadelphia, PA 19140

## Abstract

To meet the current needs of researchers who perform systematic reviews in health care settings, libraries need to provide high-quality educational services for researchers as part of their systematic review services. A team of librarians with diverse skills is also important for ensuring the growth and sustainability of systematic review services. This commentary describes a new team-based systematic review service model that can transform systematic review services by providing a pathway for librarians to offer a comprehensive educational service for systematic review research in a variety of health sciences library settings.

## BACKGROUND

Systematic reviews first appeared as an emerging area for medical librarians in the early 1990s [1, 2]. Since that time, the role of librarians in systematic reviews has continued to evolve. Librarians, while recognized for their search expertise, are also called upon for their expertise in formulating research questions, assistance in selecting the right review type, and recommendations of systematic review tools [3–6]. In recent years, articles are emerging related to systematic review services in the medical library setting [7–10].

Several different systematic review service models exist, including individual librarian–based, team-based, and fee-based models [11], which can be either formal or informal. These services primarily help researchers in conducting systematic review searches, but the level of service provided varies by institution. An individual librarian–based systematic review service model consists of a single librarian, which is a common model in a small library setting that may only have one librarian. A team-based systematic review service model preferably consists of more than two librarians and may include all reference and/or liaison librarians in a library, forming a team. While systematic review services are often provided at no charge, a fee-based systematic review service model also exists. In this model, a fee is charged for all services or for those services that are beyond conducting the search, such as help with screening references or extracting data.

One of the challenges in providing a systematic review service using any model can be the level of researcher knowledge of the systematic review process, as a low degree of researcher knowledge can lead to a librarian serving the role of “educator in addition to being the searcher,” as documented in a recent survey [12]. The author’s position as the biomedical and research services librarian at Temple University Health Sciences Library provided the opportunity to start a formal systematic review service. In the beginning, my focus was primarily on searching aspects, but I quickly learned that researchers were seeking more training about other aspects of the systematic review process. Through previous experience conducting systematic reviews as a medical researcher working outside the traditional library setting, I developed an understanding of the needs of researchers and the skills that could be helpful when conducting systematic reviews. I was able to use this experience to educate researchers seeking systematic review assistance, and soon the demand for these educational services started to grow. To respond to this increased demand, my library decided to implement a team-based approach to provide a formal systematic review service with an educational component.

This commentary describes the systematic review service model that was developed and implemented at Temple University Health Sciences Library, with the goal of making it available to other libraries to adapt and use. Rather than a one-size-fits-all approach, this model can be repurposed, reused, or remixed to give librarians the ability to implement and expand their educational systematic review services successfully. By providing a comprehensive, transparent, and open model, librarians can easily adapt the materials from the model that they need to build or expand their systematic review services.

## OVERVIEW OF THE MODEL

**Figure 1:**
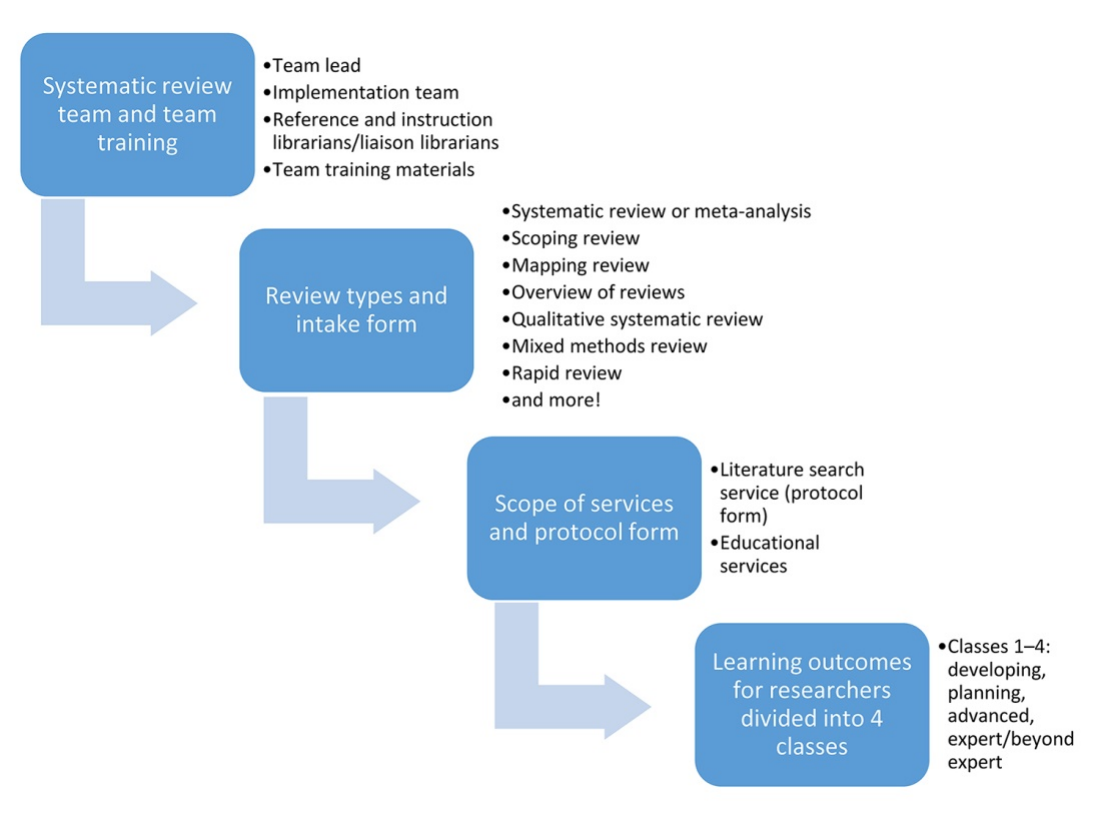
Team-based systematic review service model

This systematic review service model focuses on supporting librarians in meeting the increasing needs of researchers for assistance and guidance during all stages of the systematic review process. The Institute of Medicine (now, the Health and Medicine Division of the National Academies of Sciences, Engineering, and Medicine) recommends that a researcher “work with a librarian or other information specialist trained in performing systematic reviews to plan the search strategy” [13]. This new systematic review service model follows and expands on this recommendation by giving researchers several options: (1) have a librarian perform the search, (2) learn how to perform the search themselves in consultation with a librarian, or (3) learn more about systematic reviews and the various steps required to complete one. The model supplements systematic review searching services by providing high-quality education and training related to the systematic review process to researchers by utilizing the existing skills of reference, research, and instruction librarians who teach evidence-based research, regardless of their prior experience with systematic reviews. The model has four main components: systematic review team and team training, review types and intake form, scope of services and protocol form, and learning outcomes for researchers (Figure 1).

### Systematic review team and team training

The first component of the model is the systematic review team. For this model to work, a team lead should be identified to provide leadership to the systematic review team, while also serving as an active member of the team. The team lead should be the librarian with the most experience or expertise with systematic reviews. The full systematic review team can potentially consist of all reference, research, instruction, or liaison librarians in the library. Selection of the team members may be an administrative decision with input from the team lead. It normally works well to have all reference or instruction librarians involved, but if that is not practical, administrators and the team lead can be more selective of who is on the team.

Before this systematic review service model is introduced into a library, a separate implementation team that consists of a few members of the systematic review team, including the team lead and a library administrator, is established to review the model, make any decisions regarding adapting it to meet their library’s goals, and provide input about the model prior to the chosen implementation date. This implementation team also helps evaluate the systematic review service after implementation and is led by the systematic review team lead. Support from administration is key for the systematic review service, and a library administrator can serve on the implementation team, systematic review team, or both.

To support the formation and training of the systematic review team, the model includes a slide deck and hands-on training materials that the team lead can use or adapt to train the other librarians on the systematic review team.

### Review types and intake form

The second component of the model focuses on the types of reviews that can be conducted. The systematic review service model includes all review types that use a comprehensive search and transparent methods (i.e., systematic review/meta-analysis, rapid review, scoping review, mapping review, overview of reviews, qualitative systematic review, mixed methods review, network meta-analysis). This expands the service to include and provide guidance on additional review types that are closely related to the systematic review (Figure 1). The model does not include narrative literature reviews; however, if a request is received for a literature review, it should be screened for the service, in case a more comprehensive review type is needed. The tool used to screen all requests, including those for narrative literature reviews, is called the intake form and is provided with the model.

### Scope of services and protocol form

The third component of the model defines the scope of services that are provided as part of the systematic review service. This model includes two distinct services: (1) the systematic review literature search service and (2) the systematic review educational service.

The systematic review literature search service includes advising on protocol development and/or registration, recommending systematic review tools to help with the review workflow, guiding what needs to be reported (e.g., PRISMA flow chart), developing the comprehensive search using multiple sources (i.e., databases, grey literature, trial registries), keeping a record of the search strategies, removing duplicate results, providing education on hand-searching, and writing the search methods section.

This service is designed for health sciences faculty, students, or staff who are interested in publishing a systematic review. Students (typically graduate students or residents) must be working with a faculty member to use this service. To be eligible for the service, requesters must complete the protocol form, schedule a meeting with a librarian, and have a minimum of two reviewers. The protocol form was developed to help process this type of request and is included with the model. I recommend that librarians make the protocol form available through a comprehensive systematic review library guide or website that can also contain information about the systematic review service and educational resources. In addition to completion of the protocol form, librarians should encourage the registration of a systematic review protocol using the PRISMA-P extension statement for guidance [14].

The systematic review educational service offers beginner-to-advanced training in systematic reviews and systematic review searching, and education can be provided in a variety of formats from individual to small groups or class settings, including flipped or distance learning instruction. Educational consultations may be drop-in visits to the library or by appointment. Educational consultation requests do not require completion of the protocol form; people seeking this service may contact the library or their specific liaisons.

The educational service is designed for health sciences faculty, students, or staff who are interested in creating or publishing a systematic review. In general, students (mostly graduate) working on a dissertation, thesis, or class assignment would be eligible for this service but not the systematic review literature search service, since they are focused on learning how to conduct systematic reviews. However, an exception is made for students who are interested in publishing and are working with a faculty member, thereby, making these students eligible for the literature search service. To enhance the quality of educational services, a set of learning outcomes for researchers was developed to use with the intake form to help librarians identify key areas where a researcher may need additional systematic review education or training.

### Learning outcomes for researchers

The last component of the systematic review service model is the learning outcomes for researchers. These learning outcomes identify various components of the systematic review process that researchers may want to learn more about. Identifying learning outcomes helps guide the librarian, especially when researchers are new to the systematic review process and do not have a full understanding of the steps needed to perform a systematic review. The learning outcomes for researchers described in this model can assist librarians in recommending education or training that the researcher will need to complete a systematic review or similar review type. These learning outcomes are intended for traditional systematic reviews that compare two interventions or treatments. Since other review types follow a similar methodology to systematic reviews, basic training in systematic reviews is still recommended as the foundation to learning the other review types. There are currently thirty-four learning outcomes included in the model, divided into four classes of increasing proficiency: developing skills, planning skills, advanced skills, and expert/beyond expert skills (Table 1).

**Table 1:** Four classes of systematic review learning outcomes for researchers

Class	Systematic review learning outcomes	Librarian role
1: Developing skills	Class 1 includes developing skills for systematic review research and requires directional skills by the librarian. The researcher is at the beginning stage of the systematic review process and needs help formulating a question, understanding the steps of a systematic review, using tools for managing citations, or locating standards.	The role of the librarian is to direct the researcher to the information they need.
2: Planning skills	Class 2 includes planning skills for systematic review research and requires directional skills by the librarian. The researcher is at a stage of the systematic review process where they feel ready to create a protocol or plan the review.	The role of the librarian is to prepare the researcher for the next stages of the review.
3: Advanced skills	Class 3 includes advanced skills for systematic review research and requires demonstrative and expert searcher skills by the librarian. The focus is mainly on the search and study selection. This class relies heavily upon expert searching skills.	The role of the librarian is to demonstrate these steps, which may require multiple consultation sessions. Note: Librarians must be willing to learn and acquire these skills because they may fall outside the typical role of the library/librarian.
4: Expert/beyond expert skills	Class 4 includes expert or beyond expert skills for systematic review research and requires demonstrative skills and/or referrals made by the librarian to a nonlibrarian with more expertise in a particular area (e.g., referral to a biostatistician for assistance with meta-analysis). Class 4 skills are more analytical and require interpreting results. This may also include incorporating advanced concepts or tools or involve future technologies or methodological approaches.	Note: Librarians must be willing to learn and acquire these skills because they may fall outside the typical role of the library/librarian. In some cases, a referral might be the only service a librarian can provide.

#### Selecting classes of learning outcomes

Each class of learning outcomes provides researchers with a different set of skills and requires different expertise on the part of the librarians providing the educational services. Some learning outcomes can be fulfilled by librarians with minimal systematic review training or experience (classes 1 and 2: developing skills and planning skills), while others require more extensive systematic review skills (classes 3 and 4: advanced skills and expert/beyond expert skills). Meeting the needs of researchers who want to develop skills in class 3 (advanced skills) requires expert searching skills, while meeting researcher needs in class 4 (expert/beyond expert skills) may entail finding institutional or community partners to provide education in the systematic review process and offering referrals for these services that extend beyond the typical roles of librarians.

#### Supporting classes of learning outcomes

After receiving training from the systematic review team lead, individual librarians can select the classes of learning outcomes that they feel most comfortable supporting. Because the classes are ordered in increasing proficiency, a librarian choosing to support a higher class of learning outcomes should also be comfortable supporting the classes below it. For example, a class 3 librarian would also support classes 1 and 2. Most librarians will select either class 1 or 2. While there may be librarians who can support class 4 themselves, with or without additional training and support, this is not expected, and it is likely that few librarians will start at class 4. Librarians can decide to support higher classes as they gain more competence in systematic reviews. If a librarian is undecided between two classes, they should select the higher class, as they will most likely be able to acquire these skills over time with practice and further training.

For a successful systematic review educational service, I recommend that libraries have at least one librarian with confidence in supporting class 3 learning outcomes. This librarian can also locate referrals for class 4 learning outcomes and serve as the systematic review service team lead. As team lead, they can also help support the team members in achieving their personal educational goals and developing skills to support varying levels of educational services.

## APPLICATION OF THE SYSTEMATIC REVIEW SERVICE MODEL

This systematic review service model is designed for the academic health sciences library setting and can be adapted to create, transform, and expand a systematic review service. The adaptability of this model and the forms allows librarians to create a custom service that is unique to their users with little time spent on development.

While the complete model may be difficult for a solo librarian to adopt, there are still components of it that can be used (i.e., learning outcomes, protocol form). Librarians in very large library settings may be able to adopt the full model with few changes. For a library with much systematic review expertise, the role of the team lead might be shared or class 4 skills could be further developed and expanded upon. For libraries with little systematic review expertise, it may be possible to hire or train a librarian who can serve as the team lead and train other librarians further down the road.

My experience creating and implementing this model at Temple University Health Sciences Library has been very successful, and I have had the chance to see firsthand the impact that it has had on both our librarians and our researchers. Since its implementation in the fall of 2017, our systematic review service team has grown from just two systematic review librarians to eleven librarians who support systematic reviews. As such, I no longer have to answer every systematic review request, which has allowed me opportunities to further my systematic review training and to train other librarians in workshop and individual settings. Feedback from librarians participating in systematic review teams has been very positive overall. We have also seen an increase in the number of educational and literature search requests, and there is now more variety of review types being completed.

However, our biggest impact cannot be measured by numbers. I have seen changes in the level of work performed by our graduate students, who have said that without this service, they would have never been able to do this type of research. One student said that I was the impetus behind her thesis and that it was nice to have someone who believed that she could do a systematic review. Additionally, because of my involvement with systematic reviews on campus, my work was recognized in one of the academic programs, and I was invited to serve on a thesis committee. Instances like these make me believe that our library has moved in the right direction and that other libraries can also benefit from a very organized and structured model such as this one.

It would not be fair to say that this is the only model that works. I only know that it works well at one library. Many libraries may already have a formal systematic review model, and I encourage you to share your models. As libraries continue to share their approaches to systematic review services, we can learn how libraries can navigate and align their services to their users today and in the future.

## CONCLUSION

The complete model, “Transforming the Systematic Review Service: A Team-Based Approach to the Library Systematic Review Service Model,” is openly available for reuse and can be found on the Open Science Framework [15]. Materials that have been developed to support implementing each of these components are provided online with the model. A formal continuing education course is being created to train librarians in using and implementing this model. All training materials that are provided to attendees as part of this future course can be repurposed for their individual libraries.

This systematic review service model advances the field of health sciences librarianship by transforming the systematic review service and utilizing the reference, research, and instruction skills that librarians already possess to provide high-quality systematic review education. In this model, every librarian has value to add to the service, and it is not expected that every librarian become a systematic review expert. This model also makes a significant contribution to the field by outlining the learning outcomes that researchers may need to achieve to complete a systematic review. This model positions librarians to play a vital role in providing high-quality systematic review education to researchers. The ability to adapt and repurpose all or some aspects of this model makes it easy to implement in a variety of library settings to transform systematic review services.

## References

[1] Schell CL, Rathe RJ (1992). Meta-analysis: a tool for medical and scientific discoveries. Bull Med Libr Assoc.

[2] Mead TL, Richards DT (1995). Librarian participation in meta-analysis projects. Bull Med Libr Assoc.

[3] Dudden R, Protzko S (2011). The systematic review team: contributions of the health sciences librarian. Med Ref Serv Q.

[4] Qiu MK, Cedrone ME, Chen Y, Liu YL, Treadwell JR Advancing a librarian’s role in a rapid review: data screening.

[5] Foster M (2015). An overview of the role of librarians in systematic reviews: from expert search to project manager. J Eur Assoc Health Inf Libr.

[6] Spencer AJ, Eldredge JD (2018). Roles for librarians in systematic reviews: a scoping review. J Med Libr Assoc.

[7] Hardi AC, Fowler SA (2014). Evidence-based medicine and systematic review services at Becker Medical Library. Mo Med.

[8] Ludeman E, Downton K, Shipper AG, Fu Y (2015). Developing a library systematic review service: a case study. Med Ref Serv Q.

[9] Knehans A, Dell E, Robinson C (2016). Starting a fee-based systematic review service. Med Ref Serv Q.

[10] Roth S, Burstein K (2016). Implementing a formal systematic review service in an academic health science library setting [conference poster].

[11] Jewell ST, Foster MJ, Dreker M, Foster MJ, Jewell ST (2017). The art of puzzle solving: systematic review services. Assembling the pieces of a systematic review: guide for librarians.

[12] Nicholson J, McCrillis A, Williams JD (2017). Collaboration challenges in systematic reviews: a survey of health sciences librarians. J Med Libr Assoc.

[13] Morton S, Berg A, Levit L, Eden J (2011). Finding what works in health care: standards for systematic reviews.

[14] Moher D, Shamseer L, Clarke M, Ghersi D, Liberati A, Petticrew M, Shekelle P, Stewart LA (2015). Preferred Reporting Items for Systematic Review and Meta-Analysis Protocols (PRISMA-P) 2015 statement. Syst Rev.

[15] Roth S (2017). Transforming the systematic review service: a team-based approach to the library systematic review service model [Internet]. Open Science Framework.

